# Flavonoid-mediated biofilm inhibition and toxicological evaluation of *Atriplex laciniata* against multidrug-resistant MRSA

**DOI:** 10.3389/fphar.2025.1577052

**Published:** 2025-06-12

**Authors:** Bandar Almutairy

**Affiliations:** Department of Pharmacology, College of Pharmacy, Shaqra University, Shaqra 11961, Saudi Arabia

**Keywords:** *Atriplex laciniata*, anti-MRSA effect, biofilm inhibition, acute toxicity, hemolysis

## Abstract

Multidrug-resistant (MDR) superbugs threaten the efficacy of antibiotics, so new drug formulations from synthetic or natural sources are needed to combat antimicrobial-resistant (AMR) infections. Traditional herbs are often considered alternatives for treating AMR and MDR infections. The present study involves evaluations of the efficacy and safety of *Atriplex laciniata* aqueous (AL-Aq-Ext) and flavonoid-rich (AL-Flv-Ext) extracts against MDR MRSA strains. The efficacies of the extracts against MRSA were tested for bacterial viability and biofilm inhibition through the MTT assay, OD_600 nm_ measurements, confocal laser scanning microscopy (CLSM) for morphological observations, and amyloid-staining Congo-red phenotypic method. The safety of each extract was evaluated through comprehensive toxicological assessments, including acute toxicity, tissue biocompatibility, vital organ toxicity, and relative hemolysis. The results indicate MRSA cell viability at minimum inhibitory concentrations (MICs) of 512 μg/mL for AL-Aq-Ext and 256 μg/mL for AL-Flv-Ext. At these MICs, the extracts also exhibited bactericidal effects with zones of inhibition of 22 mm for AL-Aq-Ext and 20 mm for AL-Flv-Ext, which are comparable to the 25 mm for vancomycin. Both extracts showed more than 90% biofilm inhibition, which were confirmed through OD_600 nm_ measurements, morphological detection based on reduction in fluorescence intensities via CLSM, and phenotype by the Congo-red amyloid-staining assay. The time-kill kinetics assays indicated prolonged bactericidal effects lasting approximately 73 h against MRSA. In terms of safety, acute toxicity studies were conducted by administering MIC doses of AL-Aq-Ext and AL-Flv-Ext orally to mice over 10 d, which revealed 100% survival rates and no immediate adverse effects. Histopathological analysis of the vital organs (liver and kidneys) showed no tissue damage, confirming the absence of acute organ toxicity; hemolysis assays demonstrated no red blood cell lysis at any tested concentration, indicating excellent blood compatibility. These findings demonstrate that *A. laciniata* extracts (AL-Aq-Ext and AL-Flv-Ext) are rich in flavonoids, safe, biocompatible, and suitable for further pharmacological development, with promising potential for preclinical and clinical trials. However, the present study is limited to acute toxicity and short-term exposure evaluations; hence, future research should focus on identifying specific bioactive compounds, evaluating the long-term toxicities, studying the pharmacokinetics, assessing the efficacies in disease models, and investigating potential immunogenicity and drug interactions to fully establish the therapeutic potential of the extracts.

## 1 Introduction

Methicillin-resistant *Staphylococcus aureus* (MRSA) plays a pivotal role in both hospital- and community-acquired infections and has emerged as a major contributor to antimicrobial-resistant (AMR) infections, which are some of the most critical threats to health globally. According to global estimates, drug-resistant infections could be responsible for up to 10 million deaths annually by 2050, potentially surpassing cancer as the leading cause of mortality (Naghavi et al., 2024). The World Health Organization (WHO) has identified antimicrobial resistance as a critical public health emergency that demands urgent action (O’Leary, 2022). MRSA bloodstream infections are associated with significant morbidity and mortality, especially in immunocompromised individuals and intensive care unit (ICU) settings (Lee et al., 2018).

In response to the growing burden of antimicrobial resistance, various therapeutic strategies are being employed globally. These include the development of new antibiotics, vaccines, synthetic analogs, immunotherapeutic approaches, bacteriophage therapy, antimicrobial peptides, combination drug regimens, and use of natural bioactive compounds derived from medicinal plants (Nandhini et al., 2022; Verma et al., 2022). Among the class of natural bioactive compounds, flavonoids have emerged as promising candidates owing to their potent antibacterial, antioxidant, and biofilm-inhibitory properties (Górniak et al., 2019; Bangar et al., 2023; Rodríguez et al., 2023). Biofilm formation by MRSA is a major challenge to treatment as it significantly reduces the efficacies of conventional antibiotics, leading to persistent and recurrent infections. Therefore, targeting biofilm formation has become an essential strategy for combating MRSA infections (Chi et al., 2022; Zhang et al., 2022).

*Atriplex laciniata* L. is a halophytic plant belonging to the Chenopodiaceae family and is commonly known as saltbush; it has been traditionally used in the treatment of various ailments, including liver disorders, infections, and metabolic diseases. Phytochemical investigations have revealed that *A. laciniata* is rich in secondary metabolites, such as flavonoids, phenolics, saponins, and terpenoids, which are known for their diverse biological activities (Kamal et al., 2015); its neuroprotective, antioxidant, anticholinesterase, insecticidal, and anthelmintic effects are well-documented (Kamal et al., 2017). Moreover, *Atriplex* species have been reported to have antibacterial, antiviral, antidiabetic, and anticancer properties, which further support their therapeutic potential (Ali et al., 2021). These effects are linked to the presence of secondary metabolites, such as terpenoids and flavonoids. Traditional medicine systems have used Atriplex species for treating diabetes, jaundice, liver disorders, and infections, reinforcing their pharmacological significance (Ali et al., 2021). The present *in vitro* studies and acute toxicity findings are used to elucidate the antimicrobial activity of *A. laciniata*, including evidence of its effectiveness against Gram-positive pathogens. Specifically, flavonoid-rich fractions from *A. laciniata* have demonstrated promising antibacterial activities, including robust inhibition zones against MRSA clinical isolates, suggesting a potential mechanism involving disruption of bacterial cell membrane and inhibition of biofilm synthesis. These findings are aligned with the broader research trend of investigating flavonoid-mediated antibiofilm effects as a novel approach for tackling resistance in MRSA strains. Despite its traditional usage and emerging scientific interest, the anti-MRSA potential of *A. laciniata*, particularly in the context of flavonoid-mediated biofilm inhibition, remains largely unexplored. Furthermore, there is limited data on its toxicological profile, which is critical for validating its safety in future therapeutic applications. By integrating natural product pharmacology with antimicrobial resistance research, the present study seeks to contribute to the growing need for plant-derived polyphenolic compounds as alternative therapeutic agents for combating multidrug-resistant (MDR), AMR, and extended drug-resistant superbugs.

## 2 Materials and methods

### 2.1 Chemicals and reagents

MTT assay reagents (Promega Corporation, United States), Congo red (AGFA, Germany), tryptic soya agar (TSA) and glucose (both from Sigma-Aldrich, United States), vancomycin (Meilun Biologics, Dalian, China), and tryptic soy broth (TSB; Sigma-Aldrich, United States) were obtained from Beyotime Institute of Biotechnology (Shanghai, China); 96-well culture plates were obtained from Corning Company (New York, United States).

### 2.2 Plant identification, collection, and drying

According to Dr. Kamal, the *A. laciniata* (L.) plant was collected during the months of May and June 2023 from ring road, Peshawar, Khyber Pakhtunkhwa, Pakistan. The plant was identified by a botanical taxonomist Dr. Muhammad Imran (Department of Botany, Shaheed Benazir Bhutto University, Sheringal, Pakistan), and the plant specimen was deposited at the herbarium of the same university (voucher no: 1014 ZK-SBBU/2023).

### 2.3 Deionized water extraction

**FIGURE 1:**
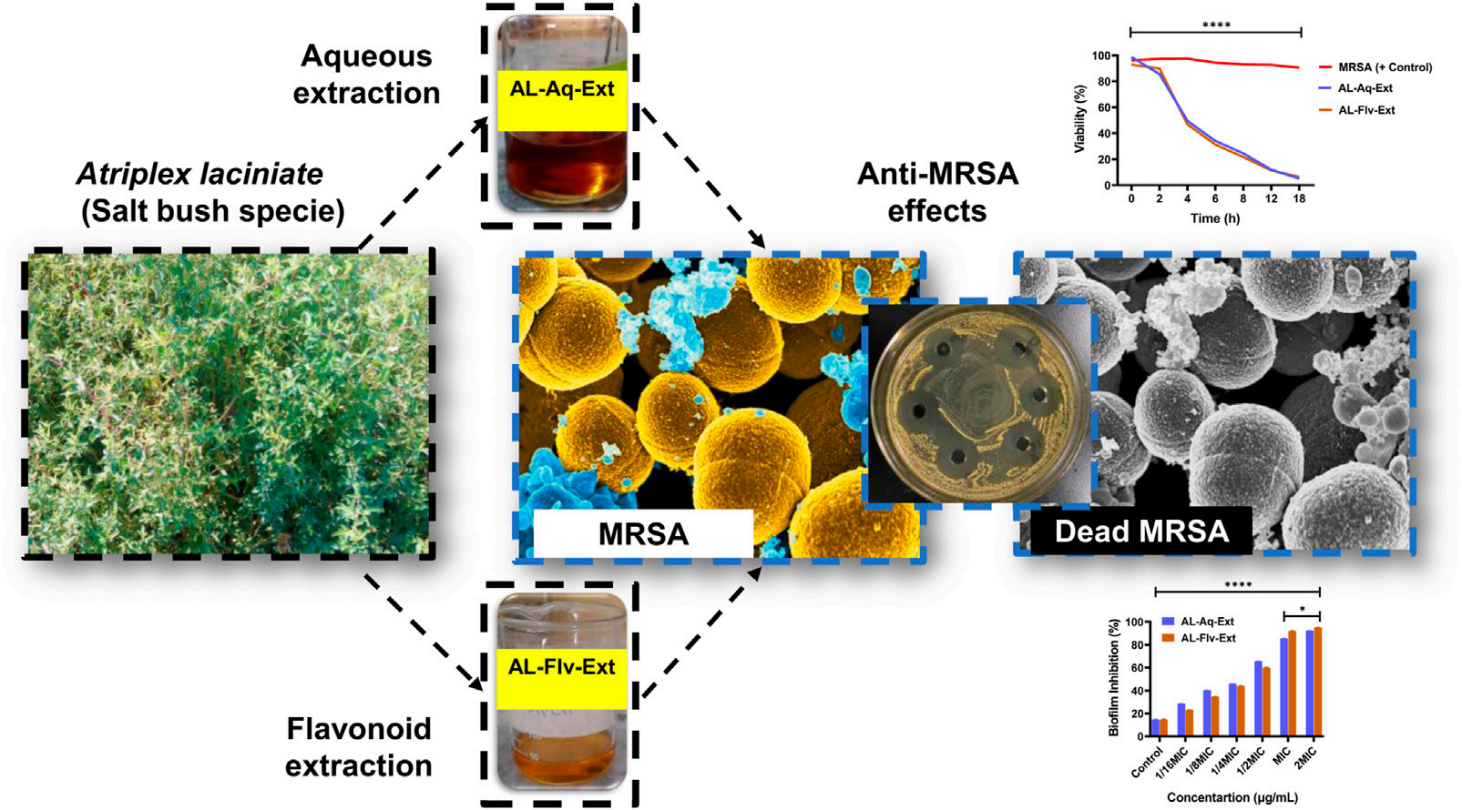
Graphical illustration showing *A. laciniata* (L.) and its aqueous (AL-Aq-Ext) as well as flavonoid-rich (AL-Flv-Ext) extracts for antibacterial actions against MRSA.

The dried plant parts were ground into a fine powder at high speed for a minimum of 8 min. Then, approximately 5 g of the fine powder was added to approximately 40 mL of deionized water and continuously sonicated at 20°C for 30 min; the sample was then maintained in a shaking incubator at 200 rpm and 37°C for 40 min. To remove any particle matter, the extracted material was vacuum-filtered twice through Whatman filter paper No. 1. The newly obtained aqueous extract (AL-Aq-Ext) was then used in all subsequent experiments (Figure 1).

### 2.4 Flavonoid extraction and total content

Total flavonoids from the crude *A. laciniata* dried plant can be quantitatively obtained using ultrasonic-assisted extraction with 70% methanol by modification of a previously reported method (Kamal et al., 2015; Chen et al., 2022; Kannan et al., 2023). Briefly, the dried powder of *A. laciniata* (5 g) was mixed with methanol and sonicated for 30 min before being filtered and concentrated using a rotary evaporator to obtain the flavonoid-rich extract (AL-Flv-Ext; Figure 1). The total flavonoid content (TFC) was then quantified using the AlCl_3_ colorimetric method; here, 0.3 mL of the plant extract, 0.15 mL of 0.5 M NaNO_2_, 3.4 mL of 30% methanol, and 0.15 mL of 0.3 M AlCl_3_·6H_2_O were added to a 10-mL test tube and mixed thoroughly. After 5 min, 1 mL of 1 M NaOH was added, and the absorbance of the mixture was measured at 415 nm. For the standard curve, rutin standard solution (0–100 mg/L) was used to determine the total flavonoid content in terms of milligrams of rutin equivalent (mg RTE/g) of the sample. The flavonoid yield (mg/g) was then calculated using the Equation 1:

$$TFC\;\left(\%Yield\right)=\frac{\text{cV}\;}{m,}$$ (1)

where *TFC* is the total flavonoid content (mg RTE/g) of *A. laciniata*, *c* is the concentration of rutin obtained from the calibration curve (mg/mL), *V* is the volume of the extract (mL), and *m* is the mass of *A. laciniata* extract (g). The percent yield (%) was obtained as follows:

$$Percent\;yield\;\left(\%\right)=\frac{Total\;flavonoid\;extracted\;\left(mg\right)}{Total\;sample\;used\;\left(mg\right)}\times 100.$$

### 2.5 Anti-MRSA effects

#### 2.5.1 MRSA culture

A fresh MRSA sample was collected from an infection site and cultured in TSA that was then cultured in TSB. Fresh cultures were used in all anti-MRSA experiments.

#### 2.5.2 Mouse model for acute toxicity

Specific-pathogen-free male SD mice were used in the acute toxicity studies and were purchased from the National Institute of Health (NIH). The animals were acclimated for 2 weeks before the experiments and fed a standard laboratory diet.

#### 2.5.3 Ethical approval

The animal experiments were carried out in accordance with the Guidelines for the Care and Use of Laboratory Animals based on the certificate issued by the Institutional Research Ethical Committee of Shaqra University, Shaqra, Saudi Arabia.

#### 2.5.4 Minimum inhibitory concentration (MIC) and minimum bactericidal concentration (MBC)

The MICs of the AL-Aq-Ext and AL-Flv-Ext samples against MRSA were determined by the microtiter plate method using 96-well plates. After determining the MIC, the MBC was evaluated by subculturing approximately 20 µL of the samples from wells showing no visible growth onto sterile TSA plates. These inoculated plates were incubated at 37°C for 24 h. The lowest concentration of the test compound that yielded no bacterial growth on the TSA surface was recorded as the MBC, which indicates ≥99.9% bacterial kill. Then, bacterial suspensions were prepared from cultures grown overnight, and the turbidity of each suspension was adjusted to an optical density at 600 nm (OD_600 nm_) of 0.7 (≈10^9^ CFU/mL). Two-fold serial dilutions of the tested samples were prepared in 100 μL volume of TSB supplemented with 1% glucose in each well. Similarly, 40 μL of fresh TSB along with 1% glucose was added to each well, followed by 60 μL of the bacterial suspension. This resulted in the final inoculum of 6 × 10^7^ CFU/mL in each well. The final concentrations of the tested samples ranged from 0.5, 1, 2, 3, 4, 8, 16, 32, 64, 128, 256, 500, 512, and 1,000 to 1,024 μg/mL. The OD absorbance measurements were performed at 595 nm using a multifunctional microplate reader.

### 2.6 Zone of inhibition (ZOI)

The ZOI refers to a circular region around the antibiotic site that prevents the growth of bacterial colonies. The ZOI is an effective technique for detecting how bacteria respond to antibiotics. The sensitivities of the AL-Aq-Ext and AL-Flv-Ext samples against MRSA (cultured on TSA plates and grown in TSB with OD_600 nm_ = 0.5) were determined using the agar well diffusion method. Fresh agar plates (30 mL) were filled with 200 µL of the MRSA suspension and allowed to cool at room temperature. Next, using a sterile cork borer, wells of diameter 6 mm and spaced part by 3 cm were generated in each plate. Subsequently, different concentrations of the samples AL-Aq-Ext and AL-Flv-Ext were added in a clockwise manner, starting at 10 μL and increasing to 20 μL, 50 μL, 75 μL, and 100 μL. The last well was established as a negative control using a sterile syringe, and the mixture was allowed to diffuse at room temperature. The plates were incubated at 37°C for 18–24 h. Using a ruler with variable diameter markings on one side, the diameter of the inhibitory zone was measured in millimeters. The ZOIs were also identified visually around each well. The entire process was performed in a laminar hood to avoid contamination.

### 2.7 Biofilm inhibition

The effects of AL-Aq-Ext and AL-Flv-Ext were also examined on preformed biofilms. The biofilms were prepared by inoculating the suspension of MRSA into the wells of a polystyrene microtiter plate, as mentioned previously. After incubation at 37°C for 24 h, the culture supernatant from each well was decanted and planktonic cells were removed by washing the wells with phosphate-buffered saline (PBS) at pH 7.2. Two-fold serial dilutions of AL-Aq-Ext and AL-Flv-Ext were prepared in TSB, and 100 μL of each dilution was added to the biofilms in the wells. The plate was further incubated at 37°C for 18 h. Then, the biofilm was fixed, stained, and quantified as described above. The following formula was used to calculate the percentage of biofilm inhibition:

$$\%\text{ Biofilm inhibition}=\frac{\text{OD}\;\text{in}\;\text{control}-\text{OD}\;\text{of}\;\text{tested}\;\text{samples}\;}{\text{OD}\;\text{in}\;\text{control}}\times 100\%$$

#### 2.7.1 Confocal laser scanning microscopy (CLSM)

MRSA was grown in TSB at 37°C for up to 24 h. Drops of the diluted culture of the bacterial strain having a density of 10^5^ CFU were applied on previously prepared glass disks, dried at ambient temperature, and subjected to CLSM analysis for the morphological investigation. The morphological differences in the bacterial strain with and without AL-Aq-Ext and AL-Flv-Ext were noted. For CLSM analysis, the disk was placed on a corning plate and stained with acridine orange.

#### 2.7.2 Congo red phenotypic method

Congo red agar (CRA) was used in a phenotypic method developed by Freeman et al. (1989) to screen the MRSA biofilm formation (Kaiser et al., 2013). Briefly, approximately 37 g/L of brain heart infusion broth supplemented with 50 g/L of sucrose, 12 g/L of agar, and 0.8 g/L of Congo red (AMERCO, Solon, OH, United States) was used here. Congo red was prepared as a concentrated aqueous solution and autoclaved at 121°C for 15 min separately from the remaining constituents; it was then cooled to 55°C and added to the mixture. The plates were inoculated and incubated aerobically at 37°C for 24–48 h. A morphology of black crystalline colonies confirms biofilm formation of MRSA against AL-Aq-Ext and AL-Flv-Ext, whereas pink colonies indicate that the biofilm formation was inhibited (Ansari et al., 2014).

### 2.8 Time-kill assay kinetics

The viability of bacterial kinetics at the MICs were determined at time intervals of 0, 0.3, 1, 2, 4, 6, 8, 12, and 18 h. The bacterial culture in the log phase was diluted with fresh medium, and approximately 20 μL of the bacteria at OD_600 nm_ of 0.5 McFarland standard (equivalent to 1.5 × 10^6^ CFU/mL) was added to the 96-well plate. Then, PBS, AL-Aq-Ext, and AL-Flv-Ext were added to the 96-well plate separately for overnight culture before being measured for OD _600nm_ with a multifunction microplate reader. The antibacterial effects were also shown by the dilution coating TSA plate method. The MRSA suspension from the well was diluted by a factor of 10^3^ using 1×PBS, and 100 μL of the diluted solution was applied to the TSA plate before culturing at 37°C overnight. The CFU/mL quantification was evaluated through the colony counting method using ImageJ software v.1.52. The groups treated with PBS were used as controls, and all experiments were conducted in triplicate.

### 2.9 Toxicology

The biocompatibility and toxicology of AL-Aq-Ext and AL-Flv-Ext were investigated using tissues, blood, and vital organs from mice bodies as per published protocols.

#### 2.9.1 Acute toxicity

The method suggested by Cho et al. (2018) was used for the acute toxicity study and evaluated for vital organ safety as well as biocompatibility through hematoxylin and eosin (H&E) staining. Under the acute toxicity study, AL-Aq-Ext and AL-Flv-Ext were administered intraperitoneally in 6-week-old SD mice of weights 20–25 g. The tested mice groups were compared with the control group (untreated animals).

#### 2.9.2 Vital organ toxicity

The mice were anesthetized with pentobarbital (200 mg/kg) and euthanized after 12 h and 24 h to study the histopathological changes and observe the vital organs like kidney and liver. At the end of the experiments, the vital tissues like liver and kidneys were collected. The samples were fixed with paraformaldehyde solution (4%, PBS) and then paraffin. Finally, H&E staining was performed for microscopic observations, where the cell nuclei were stained blue while the extracellular matrix and cytoplasm were stained pink (Kamal et al., 2022).

##### 2.9.3 Hemolysis (0, 24 h)

The blood biocompatibility and safety profiles of AL-Aq-Ext and AL-Flv-Ext were evaluated through relative hemolysis. As the AL-Aq-Ext and AL-Flv-Ext were administered orally, it is important to note that they may cause hemolysis if absorbed. Briefly, 2% (v/v) red blood cells suspension was prepared in normal saline (0.9%) using fresh blood from the SD rats. Then, samples of the red blood cells suspended in normal saline (negative control), deionized water (positive control), as well as AL-Aq-Ext and AL-Flv-Ext (0.1, 0.2, 0.3, 0.4, and 0.5 mL) were added to each tube, as outlined in Table 1. Hemolysis was evaluated at 0 h and finally after 24 h. The rate of hemolysis was evaluated by retrieving the supernatant from each sample and measuring the OD_450_ _nm_ values using a multifunction microplate reader.

**TABLE 1:** Hemolysis tests on the AL-Aq-Ext and AL-Flv-Ext samples.

Groups	1	2	3	4	5	6	7
2% RBC suspension (mL)	2.5	2.5	2.5	2.5	2.5	2.5	2.5
Normal saline (0.9%, mL)	2.4	2.3	2.2	2.1	2.0	2.5	0
Distilled water (mL)	0	0	0	0	0	0	2.5
AL-Aq-Ext (mL)	0.1	0.2	0.3	0.4	0.5	0	0
AL-Flv-Ext (mL)	0.1	0.2	0.3	0.4	0.5	0	0

Key: 1–5 = 0.1-, 0.2-, 0.3-, 0.4-, and 0.5-mL samples (AL-Aq-Ext and AL-Flv-Ext), 6 = normal saline (negative control), 7 = deionized water (positive control).

### 2.10 Statistical analysis

All data were represented as mean ± standard error of the mean (SEM). Graphs were plotted and appropriate statistically significant differences were assessed by one-way and two-way ANOVA, Tukey test, Dunnetts test, Sidak’s multiple comparisons test, linear regression, and correlations using GraphPad Prism software v.8.4.2. The differences were considered to be statistically significant at *p < 0.05, **p < 0.01, ***p < 0.001, and ****p < 0.0001, with *ns* indicating non-significant difference.

## 3 Results

### 3.1 TFC

The TFCs of both the aqueous and methanolic extracts were calculated by the ultrasonic-assisted extraction method. Table 2 shows the TFCs of both the aqueous and methanolic extracts, including the total phenolic content, as reported previously (Kamal et al., 2015).

**TABLE 2:** Total flavonoid contents of the aqueous and methanolic fractions of *A. laciniata.*

Sample	Quantity (g)	Total flavonoids (mg RTE/g of sample) ± SEM	Percent yield (%)
AL-Aq-Ext	5	93.86 ± 0.46	9.386
AL-Flv-Ext	5	166.45 ± 0.93	16.645

Key: AL = *A. laciniata*, Ext = extract, Aq = aqueous, RTE = rutin equivalent in mean ± SEM (n = 3).

### 3.2 Anti-MRSA effects

**FIGURE 2:**
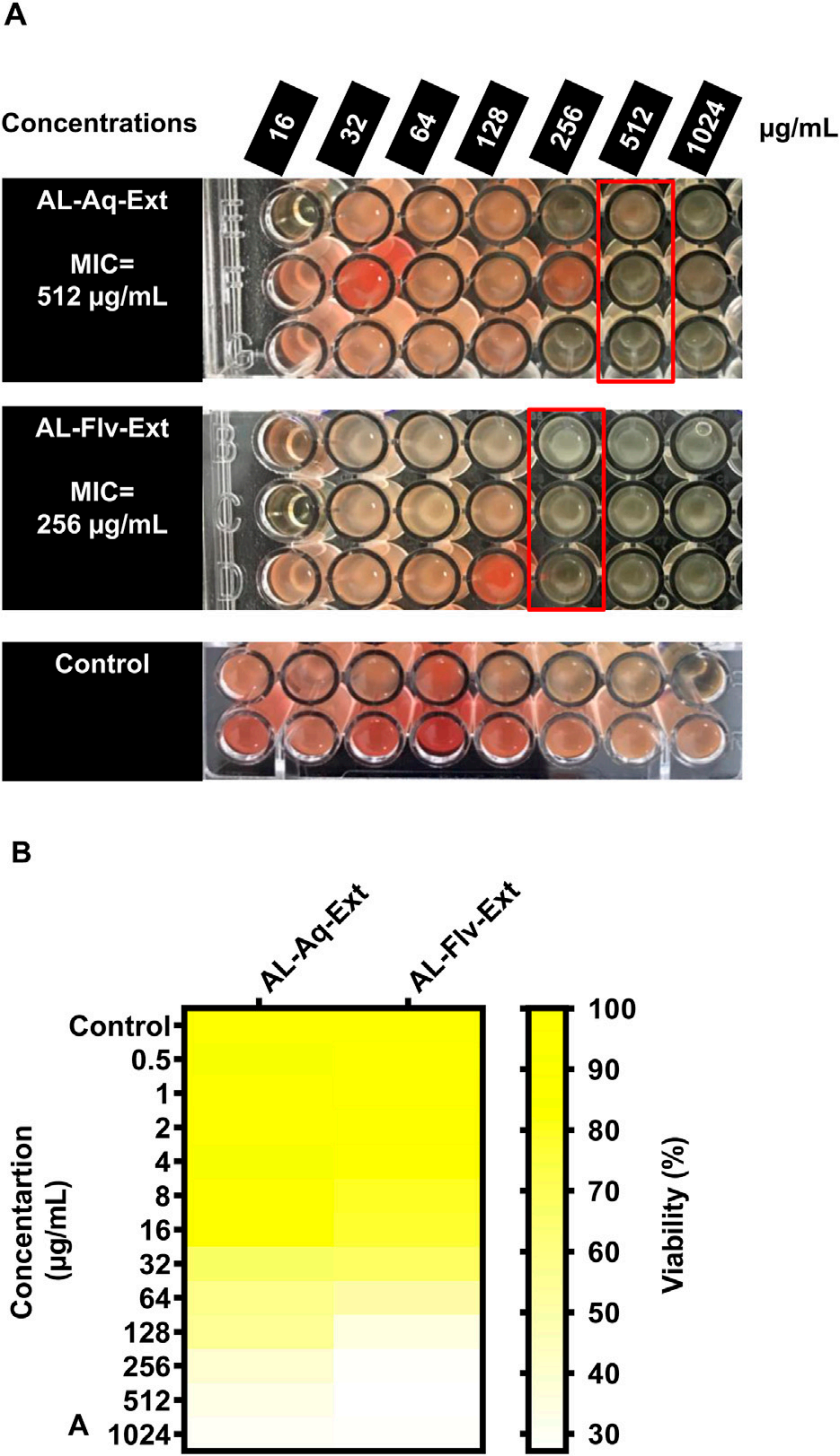
Anti-MRSA effects of *A. laciniata* aqueous (AL-Aq-Ext) and methanolic (AL-Flv-Ext) extracts based on the **(A)** MTT assay and **(B)** minimum inhibitory concentration (MIC) heatmap.

Figures 2A,B show the MICs and MTT assay results of AL-Aq-Ext and AL-Flv-Ext. The MICs obtained were 512 μg/mL for AL-Aq-Ext and 256 μg/mL for AL-Flv-Ext, indicating that AL-Flv-Ext exhibits a stronger antibacterial effect than AL-Aq-Ext. Similarly, at the same MICs, both extracts possess bactericidal effects (MBCs), showing prominent effects against MRSA.

### 3.3 ZOIs

**FIGURE 3:**
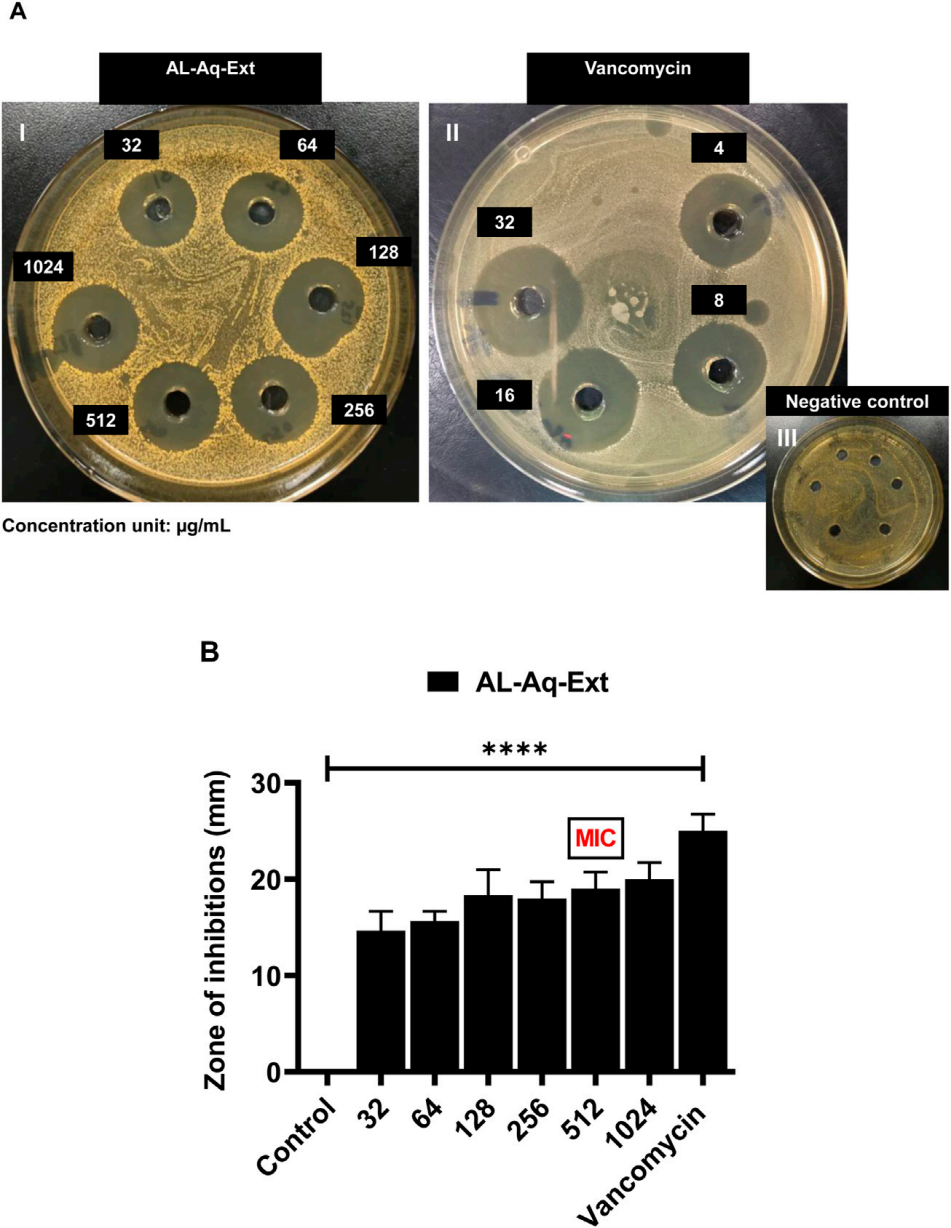
Zone of inhibition (ZOI) of *A. laciniata* aqueous extract against MRSA based on the well diffusion method. **(A)** ZOIs of **(I)** AL-Aq-Ext, **(II)** vancomycin (positive control), and **(III)** negative control. **(B)** ZOIs (mm) of selected concentrations of the aqueous extract compared to the positive and negative controls (n = 3, **p < 0.01, ***p < 0.001, and ****p < 0.0001).

**FIGURE 4:**
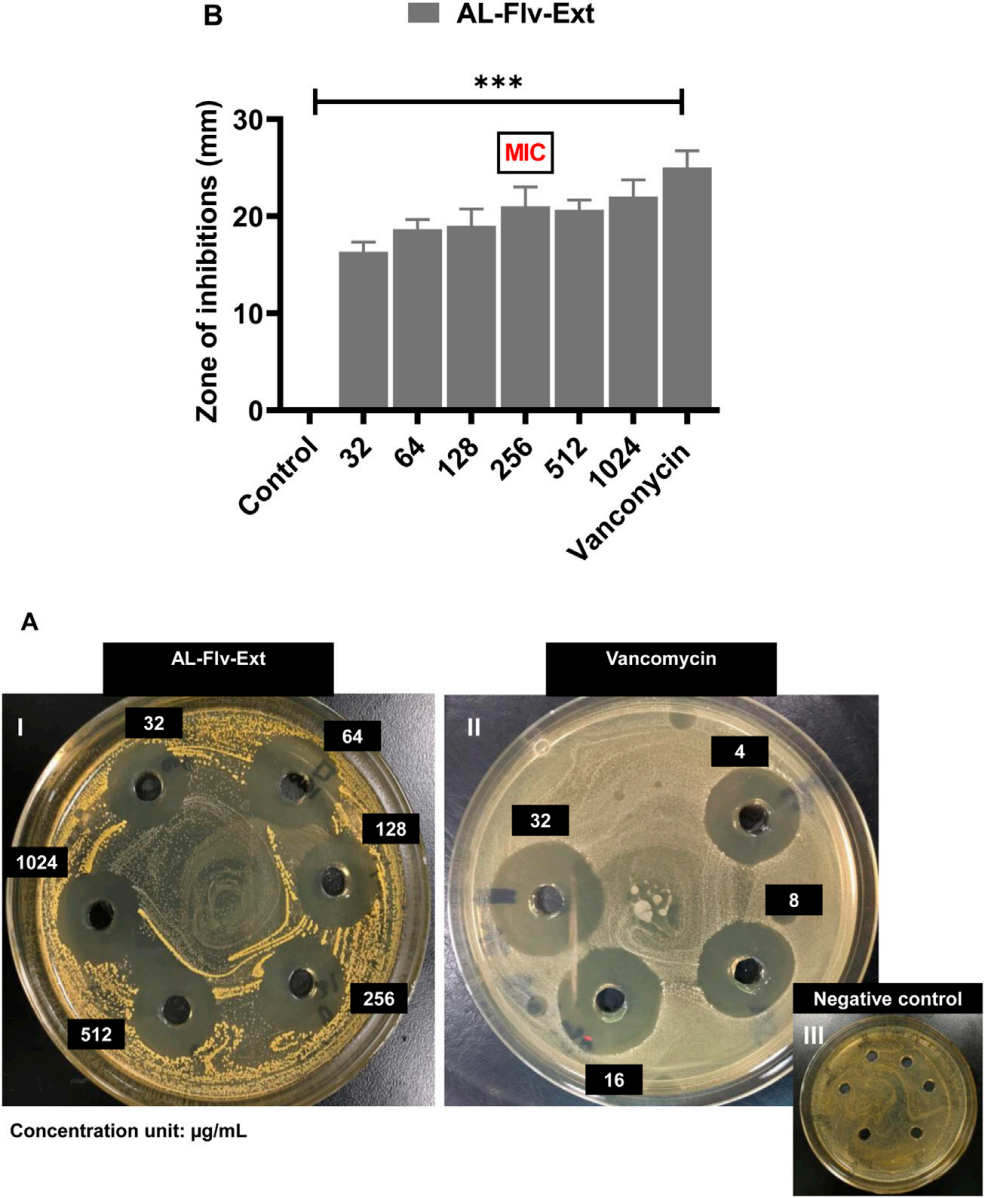
ZOIs of *A. laciniata* methanolic extract containing flavonoids (AL-Flv-Ext) against MRSA based on the well diffusion method. **(A)** ZOIs of **(I)** AL-Flv-Ext, **(II)** vancomycin (positive control), and **(III)** negative control. **(B)** ZOIs (mm) of selected concentrations of the methanolic extract compared to the positive and negative controls (n = 3, **p < 0.01, ***p < 0.001, and ****p < 0.0001).

At the MIC, AL-Aq-Ext shows a ZOI of approximately 22 mm (Figure 3AI) and is comparable to that of vancomycin (25 mm; Figure 3AII), which is a bacterial cell synthesis inhibitor considered as a last-line defense against MDR MRSA strains (Kamal et al., 2022); Figure 3AIII shows the negative control. Figure 3B shows a comparison of the ZOIs at different concentrations of the extract and for the control. Similarly, Figures 4AI–III show the ZOIs for AL-Flv-Ext at MIC (22 mm) compared to the positive control vancomycin (25 mm) as well as the negative control, respectively. Figure 4B shows a comparison of the ZOIs at different concentrations of the extract and for the control.

### 3.4 Biofilm inhibition

#### 3.4.1 OD_600_ _nm_ measurements

**FIGURE 5:**
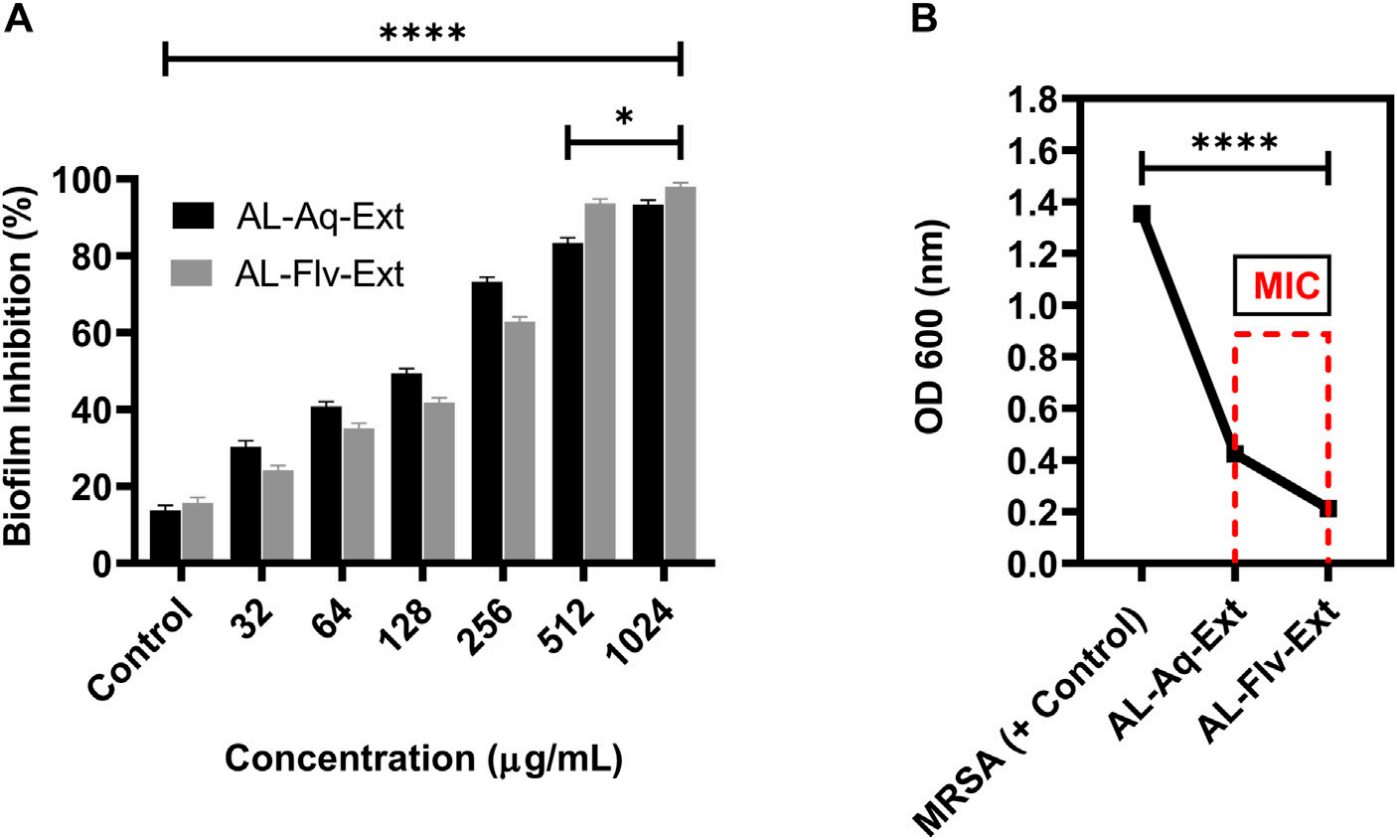
Biofilm inhibition by AL-Aq-Ext and AL-Flv-Ext against MRSA: **(A)** percentage biofilm inhibition at various concentrations; **(B)** OD_600 nm_ of MRSA biofilm inhibition by AL-Aq-Ext and AL-Flv-Ext at MICs compared to positive control (TSB-cultured MRSA) (n = 3, **p < 0.01, ***p < 0.001, and ****p < 0.0001).

Biofilm inhibition caused by the two extracts of *A. laciniata* are depicted in Figure 5A, which shows the activities of AL-Aq-Ext and AL-Flv-Ext at different concentrations, including their MICs, compared to the negative control (without extracts and standard drugs). Both extracts show inhibitions above 90% at their MICs. Figure 5B confirms biofilm inhibition through the OD_600_ _nm_ measurements at MICs for both AL-Aq-Ext and AL-Flv-Ext compared to MRSA (positive control). The optical densities were below 0.4 for both extracts, indicating biofilm disruption of MRSA (OD _600_ _nm_ = 1.4) after incubation for more than 24 h.

#### 3.4.2 CLSM measurements

**FIGURE 6:**
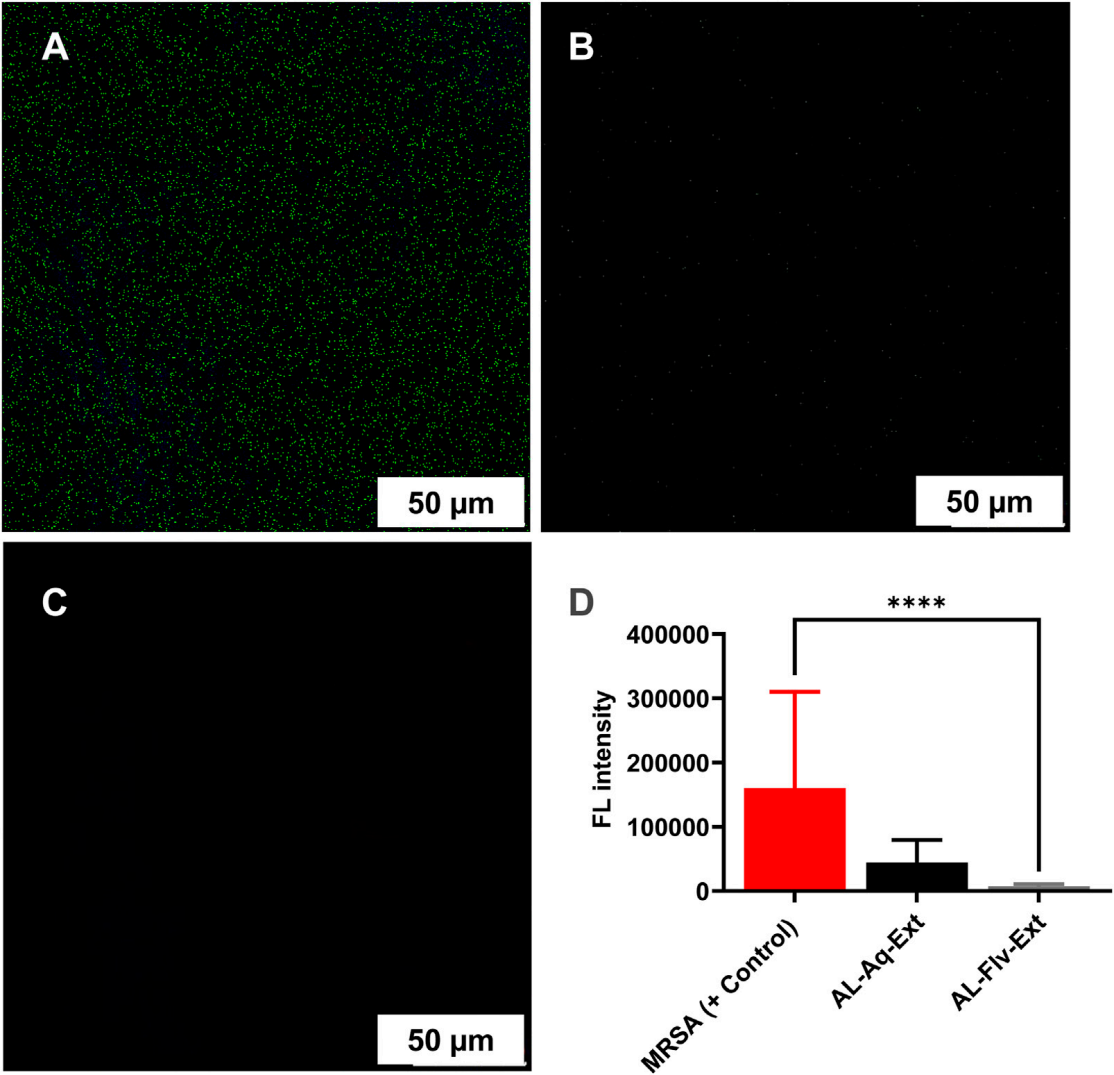
Confocal laser scanning microscopy (CLSM) observations of the anti-MRSA effects of *A. laciniata* at scale bar = 50 μm: **(A)** MRSA (positive control), **(B)** AL-Aq-Ext at MIC, **(C)** AL-Flv-Ext at MIC, and **(D)** fluorescence intensity measurements of the anti-MRSA effects of *A. laciniata* (*n* = 3, **p < 0.01, ***p < 0.001, and ****p < 0.0001).

The high intensities of green fluorescence indicates the survival of MRSA and its cytoplasmic integrity. Figure 6A shows the CLSM image of MRSA (positive control), and Figures 6B,C represent the images of MRSA treated with AL-Aq-Ext and AL-Flv-Ext, respectively. Figure 6D shows the fluorescence intensity measurements of both extracts compared to the positive control.

#### 3.4.3 Congo red phenotypic method

**FIGURE 7:**
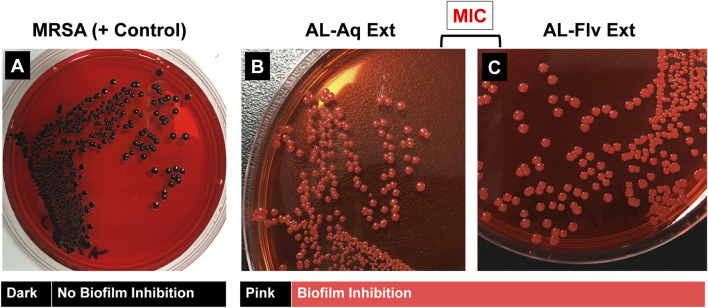
Congo red phenotypic assay for amyloid fibril staining of MRSA biofilm: **(A)** Amyloid-fibril-producing MRSA shows distinct black colonies (positive control); application of **(B)** AL-Aq-Ext and **(C)** AL-Flv-Ext at their MICs show pink-colored colonies indicative of biofilm inhibition.

The Congo red method of determining biofilm inhibition through a phenotypic approach was used to assess the efficacies of AL-Aq-Ext and AL-Flv-Ext against MRSA. Figures 7A–C show the results for MRSA (positive control), Al-Aq-Ext, and AL-Flv-Ext, respectively. The pinkish amyloid staining observed with the two extracts indicates inhibition of biofilm formation.

### 3.5 Time-kill assay kinetics

**FIGURE 8:**
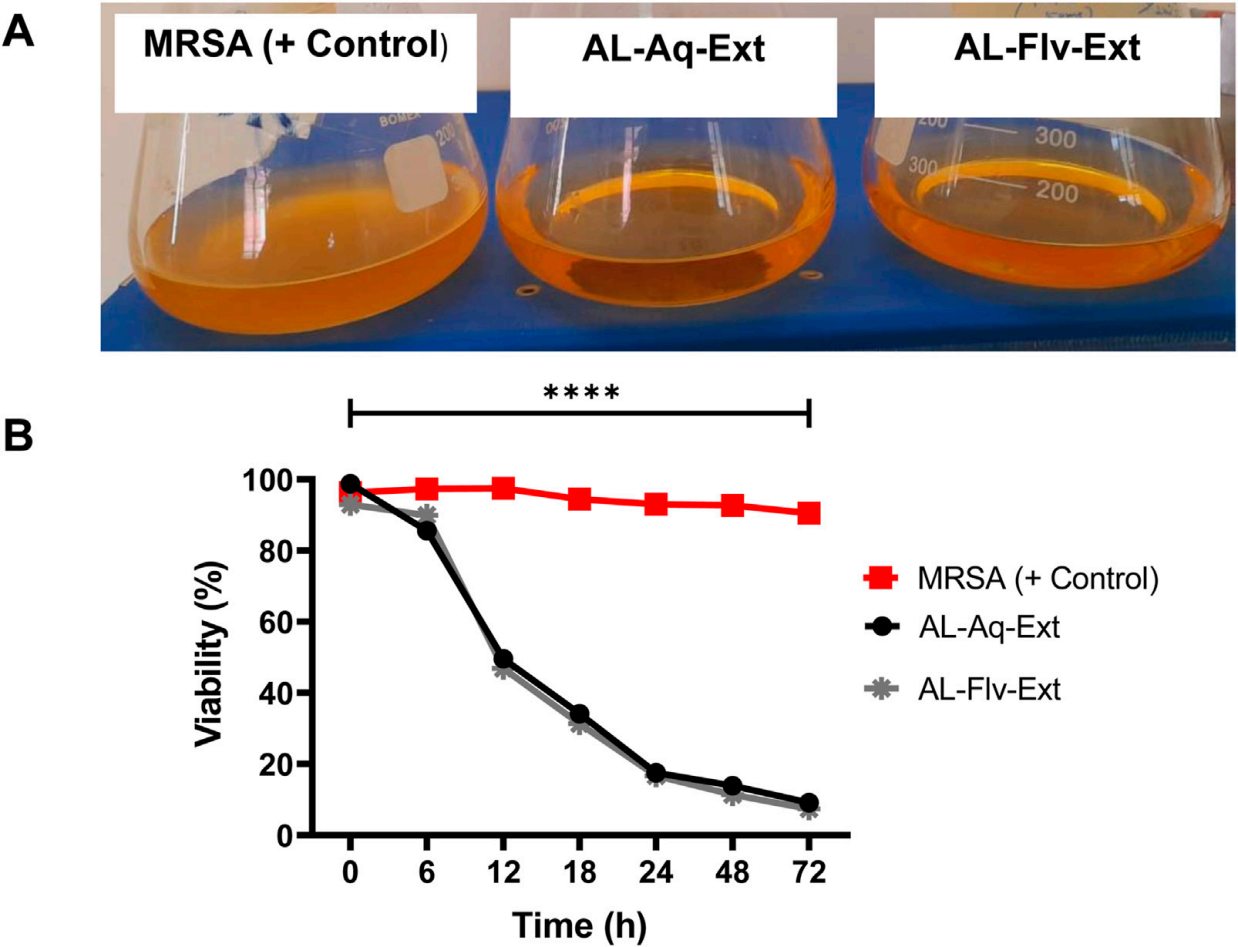
Time-kill assay kinetics of *A. laciniata* against MRSA: **(A)** turbidity observations of all samples after 72 h of incubation at 37°C and 220 rpm; **(B)** time-kill assay viabilities of AL-Aq-Ext and AL-Flv-Ext compared to MRSA (positive control) (n = 3, **p < 0.01, ***p < 0.001, and ****p < 0.0001).

MRSA cultures were treated for 72 h with AL-Aq-Ext and AL-Flv-Ext to determine the time-kill assay kinetics. Figure 8A shows the turbidity observations of MRSA (positive control without drugs), AL-Aq-Ext, and AL-Flv-Ext samples after 72 h of incubation at 37°C and 220 rpm. Figure 8B shows the time-kill assay percent viabilities of all samples at their MICs.

### 3.6 Toxicology

The safety and efficacies of the *A*. *laciniata* extracts were analyzed through toxicological and tissue biocompatibility studies.

#### 3.6.1 Acute toxicity

**FIGURE 9:**
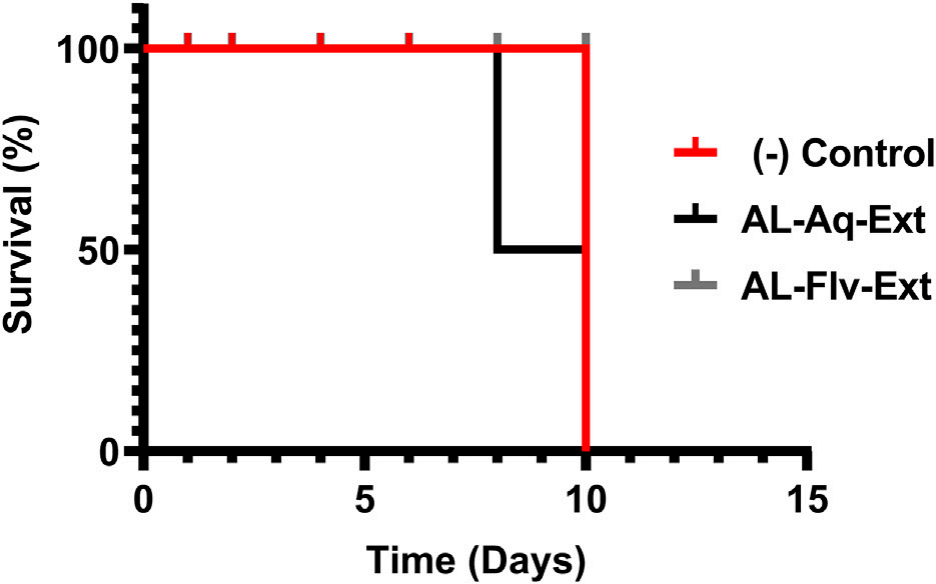
Acute toxicities (survival %) of mice treated with AL-Aq-Ext and AL-Flv-Ext compared to negative control (*n* = 6 per group, survival curves were compared through the log-rank (Mantel–Cox) test, *p* = ns).

Figure 9 shows the acute toxicities (survival %) of AL-Aq-Ext and AL-Flv-Ext compared to the negative control when administered for 10 d. Both extracts show 100% survival percentage at MICs when administered orally.

#### 3.6.2 Vital organ/tissue toxicity

**FIGURE 10:**
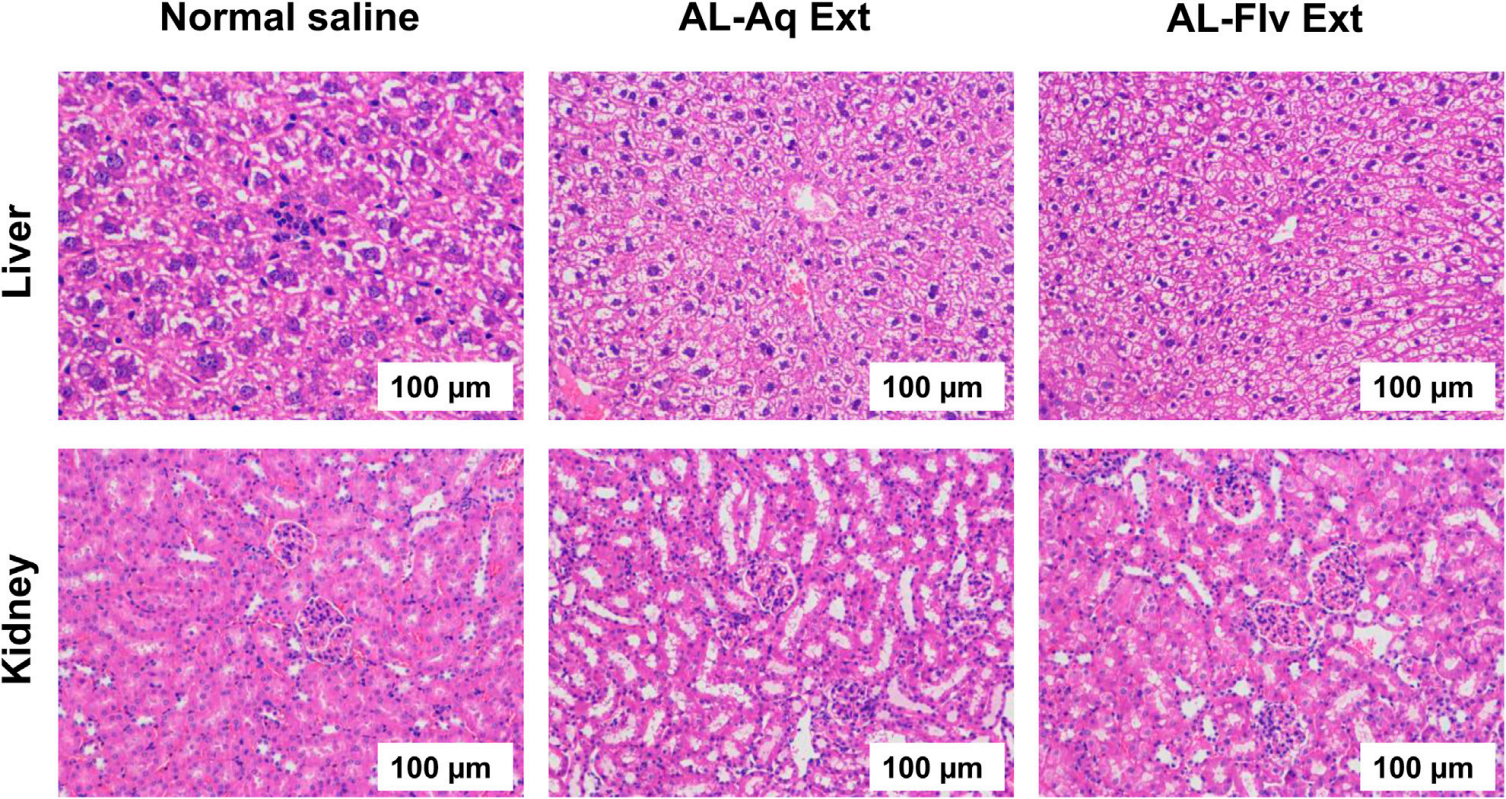
H&E-stained histological images of liver and kidney (vital organs) of mice after treatment with AL-Aq-Ext, AL-Flv-Ext, and normal saline as control (scale bar = 100 µm).

The vital organs like liver and kidneys of the study animals were tested for acute toxicity; the study animals were then anesthetized and euthanized for organ dissection. Figure 10 shows the H&E-stained histological images (scale bar = 100 µm) of the liver and kidney (vital organs) of the mice after treatment with AL-Aq-Ext, AL-Flv-Ext, and normal saline (control).

##### 3.6.2.1 Relative hemolysis

**FIGURE 11:**
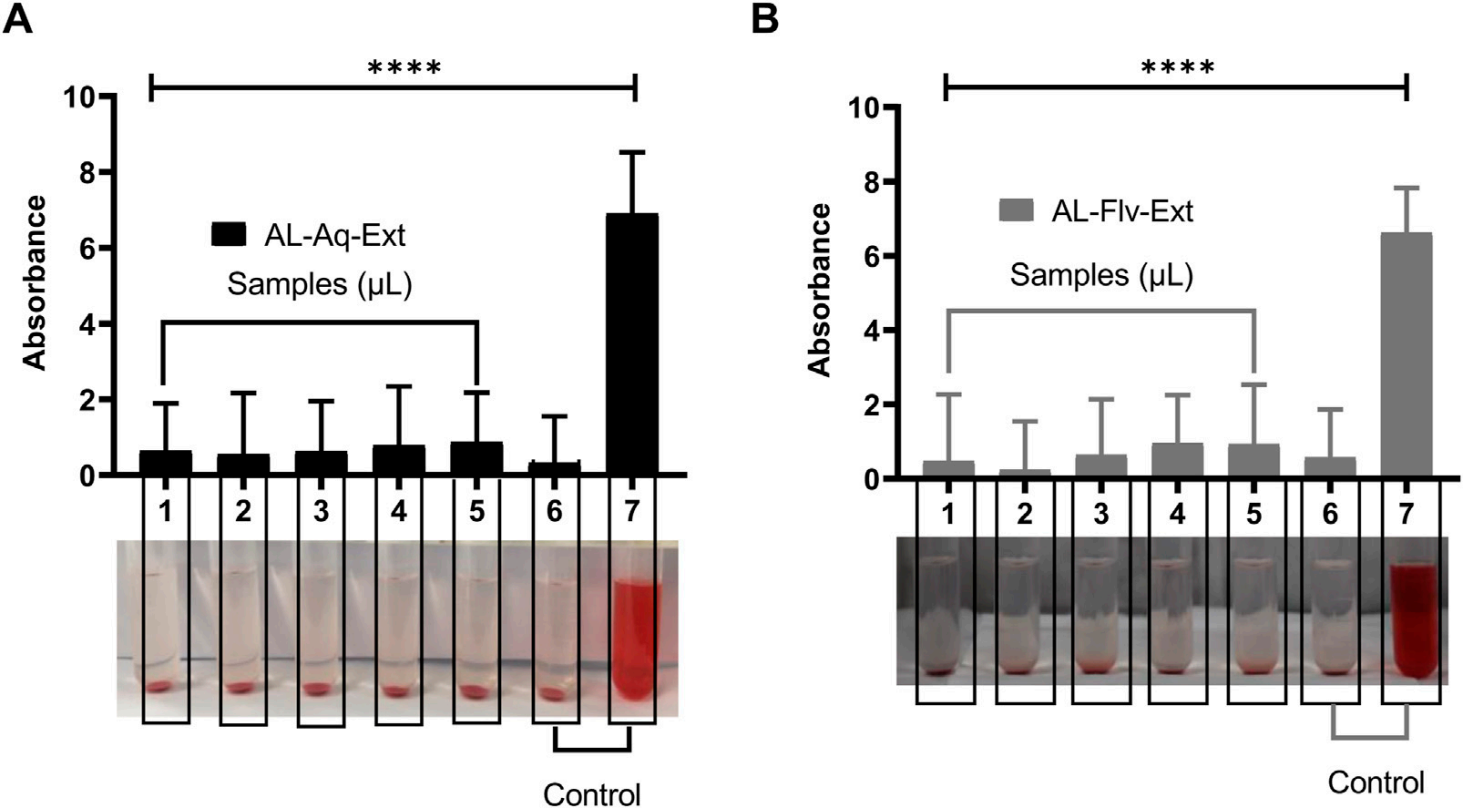
Blood compatibilities and absorbances for relative hemolysis after 24 h of observations (*n* = 8, mean ± SEM, ****p < 0.0001): **(A)** AL-Aq-Ext and **(B)** AL-Flv-Ext. Samples 1–5 correspond to concentrations of 100, 200, 300, 400, and 500 µL of AL-Aq-Ext and AL-Flv-Ext, respectively; 6 = normal saline (negative control); 7 = distilled water (positive control).

Figures 11A,B show the blood biocompatibilities and absorbances for the relative hemolysis caused by AL-Aq-Ext and AL-Flv-Ext, respectively. No relative hemolysis was observed for both extracts at any of the concentrations used, which highlight their biocompatibility with blood.

## 4 Discussion

In most of the flavonoid-rich medicinal plants, the glycosylated flavonoids are often soluble in aqueous solvents and exert anti-MRSA effects by interfering with bacterial biofilm formation as well as membrane integrity. The more lipophilic aglycone flavonoids are soluble in organic solvents and can disrupt bacterial cell and biofilm formation through their quorum sensing effects, which interrupt intracellular responses and enhance membrane permeability (Zhao et al., 2019; Chen et al., 2022; Naeem et al., 2022). In addition to their established roles in biofilm suppression, flavonoids exhibit broad-spectrum antibacterial activities through multiple synergistic mechanisms. Flavonoids disrupt bacterial membrane integrity by altering the permeability and fluidity, leading to cytoplasmic leakage and cell lysis (Wang et al., 2021; Wang et al., 2024). For instance, quercetin and catechins embed in the lipid bilayers to compromise membrane stability (Wang et al., 2023). Flavonoids further impair bacterial viability by interfering with critical metabolic pathways, such as glycolysis and ATP synthesis, thereby depleting the cellular energy reserves (Wang et al., 2020; Wang et al., 2021). Additionally, they induce oxidative stress and bind to nucleic acids, thereby inhibiting DNA replication and protein synthesis (Wang et al., 2024). These multitarget actions enhance their impartial efficacies and potentiate conventional antibiotics by facilitating intracellular drug uptake (Wang et al., 2023). Given the high content of flavonoids in *A. laciniata*, its anti-MRSA and biofilm disruption effects were explored in the present study. AL-Aq-Ext and AL-Flv-Ext were used in the experiments in this work; the higher efficacy of AL-Flv-Ext is attributed to the presence of flavonoids (glycosylated and aglycon), which are known to disrupt bacterial quorum sensing, membrane integrity, virulence factor expression, and biofilm formation (Bangar et al., 2023; Rodríguez et al., 2023). Our findings align with this expectation, suggesting that the observed antibacterial effects may arise from a combination of membrane disruption, metabolic inhibition, and nucleic acid interference. Future studies should quantify the relative contributions of these mechanisms to optimize flavonoid-based therapies.

The MIC/MBC results demonstrate the potent anti-MRSA effects of AL-Aq-Ext and AL-Flv-Ext. At their respective MICs, both extracts exhibit strong inhibitory effects on MRSA growth, which is attributable to the higher concentrations of bioactive compounds, such as glycosylated and aglycone flavonoids (Cushnie and Lamb, 2011). Furthermore, the MBC results affirm that both extracts possess bactericidal activities, suggesting their ability to not only inhibit bacterial growth but also MRSA cells. The dual inhibitory and bactericidal effects are particularly significant against MRSA, which is infamous for its resistance to conventional antibiotics (Lee et al., 2013). Reduction in MRSA viability observed through the MTT assay further supports these findings, which are aligned with previous studies highlighting the enhanced antimicrobial potential of flavonoid-rich plant extracts against MDR pathogens (Tsuchiya et al., 1996; Cushnie and Lamb, 2011). The potent anti-MRSA effects of AL-Aq-Ext and AL-Flv-Ext are promising for the development of novel anti-MRSA agents, particularly given the currently rising crisis of antibiotic resistance. However, further studies are warranted to elucidate the specific bioactive compounds responsible for such activities and to evaluate their action mechanisms in greater detail.

The potent anti-MRSA effects of AL-Aq-Ext and AL-Flv-Ext were subjected to ZOI analyses and were compared to that of vancomycin, which is used for critical bacterial process disruption. These flavonoid extracts may interfere with cell wall syntheses or membrane integrity, akin to vancomycin, which targets peptidoglycan cross-linking by binding to the D-alanyl-D-alanine residues. Previous studies suggest that the presence of flavonoids and phenolic acids in these extracts mostly contributes to their anti-MRSA efficacy, which may either disrupt bacterial enzymes, protein synthesis, or DNA replication, as well as compromise membrane potential and efflux pumps (Cushnie and Lamb, 2011). These multitarget mechanisms reduce the likelihood of developing resistance, which is a significant advantage in combating MRSA, particularly given the emergence of vancomycin-intermediate and vancomycin-resistant *S. aureus* (VISA and VRSA) strains (Gardete and Tomasz, 2014). The dose-dependent increase in anti-MRSA activity observed herein further highlights the therapeutic potential of the extracts, which could become promising alternatives to conventional antibiotics owing to their broad-spectrum activity and lower likelihood of resistance (Ayaz et al., 2019; Khare et al., 2021). However, further extensive research efforts on the safety and efficacy of these extracts can clarify their precise action mechanisms and molecular targets (WHO, 2014; Tacconelli et al., 2018; Zhou et al., 2023).

The MRSA biofilm disruption efficacies of AL-Aq-Ext and AL-Flv-Ext underscore their potential as anti-biofilm agents. At their MICs, these extracts show 90% biofilm inhibition, suggesting that the flavonoids and other bioactive compounds interfere with biofilm formation and stability, as affirmed through OD _600 nm_ measurements. Furthermore, the reduction in fluorescence intensities in the CLSM images indicates biofilm disruption and bacterial viability, which are further corroborated through Congo red phenotypic findings. The reduced amyloid staining in the Congo red experiments reflect the reduction in biofilm matrix production. Previous studies show that these flavonoid extracts mechanistically target the extracellular polymeric substances or quorum sensing pathways, which are critical for biofilm formation and maintenance (Davies et al., 1998; Costerton et al., 1999; Trebino et al., 2021; Gündog et al., 2023). The long-lasting anti-MRSA effects of *A. laciniata* extracts (AL-Aq-Ext and AL-Flv-Ext) were explicitly demonstrated through time-kill assay kinetics, which revealed the reduction in bacterial viability over 72 h. These sustained antimicrobial effects suggest that the extracts act through time-dependent mechanisms, potentially involving disruption of cell membrane integrity, interference with metabolic pathways, or inhibition of essential enzymes (Nostro et al., 2007; Gibbons, 2008; Kumar and Engle, 2023).

The toxicological evaluations of *A. laciniata* extracts (AL-Aq-Ext and AL-Flv-Ext) confirm their safety and biocompatibility while supporting their potential as future therapeutic agents. The excellent oral tolerability with 100% survival rate in the acute toxicity studies at MICs depicts the presence of low-toxicity bioactive compounds like flavonoids and polyphenols (Thakurdesai et al., 2024). Histopathological analyses of the vital organs like the liver and kidneys indicate no tissue damage, revealing the absence of acute organ toxicity. Studies suggest that these findings may be attributed to the antioxidant and anti-inflammatory properties of both AL-Aq-Ext and AL-Flv-Ext, which protect tissues from oxidative stress (Xiang et al., 2018; Majee et al., 2023). Additionally, the absence of relative hemolytic activities at all concentrations highlights the blood compatibility of these extracts, which is a critical factor for systemic administration. This is likely due to the membrane-stabilizing effects of polar phytochemicals in the extracts (Kamal et al., 2022; Aiswarriya et al., 2023; Iancu et al., 2023). Collectively, these findings suggest that AL-Aq-Ext and AL-Flv-Ext are safe and effective, with their mechanisms being rooted in antioxidant, anti-inflammatory, and biocompatible properties. Further studies should identify the specific bioactive components in these extracts, in addition to their isolation, evaluation of the long-term safety, pharmacokinetics, and mechanism-focused research. These studies should also consider the need for future phytochemical profiling using liquid chromatography to identify the individual flavonoids responsible for the observed bioactivities.

## 5 Conclusion

The flavonoid-mediated anti-MRSA effects of *A. laciniata* extracts, including biofilm inhibition, highlight their efficacy and suitability for further pharmacological developments, which can become viable alternatives for combating antimicrobial resistance. The toxicological evaluations further demonstrate their safety, biocompatibility, and potential as therapeutic agents, which are additionally supported by the absence of acute toxicity, organ damage, and hemolytic activity. The efficacy and safety of both flavonoid extracts highlight their suitability, making them promising candidates for preclinical and clinical trials. However, future research efforts should focus on identifying the specific bioactive compounds in these extracts as well as exploring their action mechanisms (e.g., efflux pump inhibition and quorum sensing). Long-term toxicity studies, pharmacokinetic profiling, as well as assessments of the chronic toxicity, immunogenicity, and drug interactions of these extracts are essential to ensure their safety for human use. Despite the promising results, the present study has some limitations with regard to the acute toxicity and short-term exposure findings; therefore, deeper investigations into the mechanisms underlying the biocompatibility and therapeutic effects are needed. In conclusion, *A. laciniata* extracts exhibit significant potential as safe and effective therapeutic agents against MRSA, justifying further exploration in both preclinical and clinical settings.

## Data Availability

The raw data supporting the conclusions of this article will be made available by the authors without undue reservation.
